# The efficacy and safety of endo‐radiofrequency for the treatment of hidradenitis suppurativa

**DOI:** 10.1111/srt.13450

**Published:** 2023-08-28

**Authors:** Elham Behrangi, Najmolsadat Atefi, Pardissadat Mireshghollah, Azadeh Goodarzi, Abbas Dehghani, Ali Zare Dehnavi, Masoumeh Roohaninasab, Sara Dilmaghani, Elaheh Lotfi

**Affiliations:** ^1^ Department of Dermatology Rasool Akram Medical Complex Clinical Research Development Center (RCRDC) School of Medicine Iran University of Medical Sciences (IUMS) Tehran Iran; ^2^ Tehran University of Medical Sciences Tehran Iran; ^3^ Yoosefabad Skin and Hair Center Tehran Iran

**Keywords:** clinical trial, efficacy, hidradenitis suppurativa, radiofrequency, safety

## Abstract

**Background:**

Hidradenitis suppurativa (HS) is a chronic and recurrent disease of the axilla and groin with inflammatory lesions. There is no definitive medication or intervention to cure the disease. Radiofrequency (RF) is a modality to destroy the lesions by transferring heat into the skin. To date, few studies have been conducted to evaluate the efficacy and safety of RF at HS.

**Methods:**

This 9‐month, prospective, nonrandomized, and single‐blinded study is a clinical trial conducted in 10 patients with refractory HS. In all patients, the initial grade of HS was evaluated. The procedure involved treating HS of the axilla with a endo‐RF wave device. Post‐treatment evaluation included: determination of the severity of the disease by a blinded dermatologist, the degree of patient satisfaction, tolerability in each patient, and complications of the procedure. We also evaluate the recurrence of the disease during a 6‐month follow‐up.

**Results:**

The satisfaction level after the intervention among under‐studied cases was excellent and good in six of cases. There was a significant difference in comparing the grading score of patients before and 3 months after receiving RF (*P*‐value: 0.01). Regarding tolerability, eight of the patients could tolerate it. We had no complication after the intervention and four cases had not recurrence during a 6‐month follow‐up.

**Conclusion:**

Endo‐RF is an effective and safe modality for treating HS however to prevent the recurrence, periodic therapy sessions are needed.

## 1 INTRODUCTION

Hidradenitis suppurativa (HS) is a chronic and inflammatory disease of apocrine glands, usually affecting the axilla and inguinal regions.^1^ This condition typically occurs after puberty and, due to its recurrent nature, has a significant effect on the patients’ quality of life and general psychological state.^2^ The primary event in the pathophysiology of HS is disruption of apocrine gland drainage and formation of comedones, leading to recurrent inflammation, infection, tissue damage, and consequently clinical lesions such as abscesses, fistulas, nodulocytic lesions, and scars.^3^ Although there is no definitive medication or modality for curing the disease, several treatments have been introduced to control HS, such as the use of topical agents, oral antibiotics, retinoids, biologic medications (TNF‐α inhibitors), and finally surgery aimed at draining or excising clinical lesions.^4^ About medical treatments, The main problem with the use of antibiotics is the development of resistance after long‐term use, variable results have been obtained with the use of oral retinoids, and biologic drugs are expensive for long‐term use.^5^ On the other hand, surgery can not be performed in a large number of patients for various reasons, such as the risk of general anesthesia and recurrences.^6^ In addition to these treatments, some interventions have been used for this disease, such as the use of ablative CO2 laser and electrosurgery, but these methods are not comfortable for the patients and it takes a long time to get a therapeutic response.^7^, ^8^ After evaluating the studies, it is necessary to consider a new and effective treatment method that is tolerable for HS patients with a short recovery time. Radiofrequency (RF) in skin diseases is not only used for remodeling collagen fibers and rejuvenation,^9^ but has also been used to treat acne vulgaris by affecting the sebaceous glands and in some studies to treat HS.^3^ The therapeutic role of radiofrequency waves in HS has been reported to transfer heating into the derm and subsequent destruction of nodulocytic clinical lesions, as well as remodeling of collagen fibers, thus improving the patients’ fibrotic lesions.^5^ Since there are few studies about efficacy and safety of this intervention, it is difficult for dermatologists and patients to decide on this method. Therefore, in this study, to evaluate the efficacy and safety of this method, we used radiofrequency in the form of a monopolar device to treat 10 patients with different intensity of HS.

## 2 MATERIALS AND METHODS

### 2.1 Study population

This 9‐month, prospective, nonrandomized and single‐blinded study is a clinical trial was performed on patients aged 15–65 years referred to our university dermatology clinic from October to December 2021 with a refractory HS. All patients underwent various treatments for at least one year, including antibiotics, retinoids and biologic drugs, but were not satisfied with the outcome of the treatment. A clinical diagnosis of HS was the inclusion criteria and the exclusion criteria of the study were included of family history of bleeding or coagulant diseases, consumption of anticoagulants, platelets less than 150,000 or blood hemoglobin less than 10, the presence of active skin or systemic infection, breastfeeding or pregnancy, malignancy and history of chemotherapy or immunosupression. Regarding these, 10 patients were enrolled after providing a written informed consent.

### 2.2 Assessment methods

At the beginning of the study, patients’ demographic (gender, age and body mass index) and clinical (cigarette smoking, disease duration, drug history, and current treatment) information was prepared and recorded in the study checklist. In all patients, before the intervention, the initial grade of the disease was determined qualitatively based on HS physicians’ global assessment (HS‐PGA) that is an available method for determining the severity and extent of recovery in the disease.^5^ The system describes six stages in HS, increasing in severity on a scale from one to six. Post‐treatment evaluation of patients with a 3‐month observation period was performed on the basis of following description: (1) Determining the severity of the disease with HS‐PGA by a blinded dermatologist (second grade), (2) The level of patient satisfaction: low response (less than 30%), moderate response (30%–50%), good response (50%–70%) and excellent response (above 70%), (3) The tolerability of each patient for the procedure (yes or no), and (4) Recording the probable complications of the intervention including erythema, heat, swelling, infection, and burning of the skin. Finally, after determining the second grade, we evaluate the recurrence of the disease (return to previous grade) during a 6‐month follow up.

### 2.3 Intervention method and device characteristics

HS of the axilla was treated by a device with radiofrequency waves (Aphrodite, 4 MHZ, monopolar, 60 W and 1 KΩ). Prior to the treatment, the entire area was disinfected and dried and after anesthesia of the entry point with lidocaine, the cannula of the device was entered into the deep dermis. The device causes heating along with deep dermis, fibrous septa, and nodulocystic lesions up to 90°C and denaturation of the lesions occurs by heat transfer via a high frequency electric current. The cannula is heat isolated along its entire length, so heat transfer is done only through its tip, not reflected and the epidermis is not damaged. Patients received treatment in three sessions.

### 2.4 Data analysis

The quantitative variables were expressed as mean and standard deviation (mean ± SD) and the qualitative variables as frequency (percent), also the Stuart–Maxwell test was used to compare two qualitative variables. The significance level was considered less than 0.05. In order to data statistical analysis, SPSS software version 22 was used.

## 3 RESULTS

### 3.1 Basic characteristics

**FIGURE 1:**
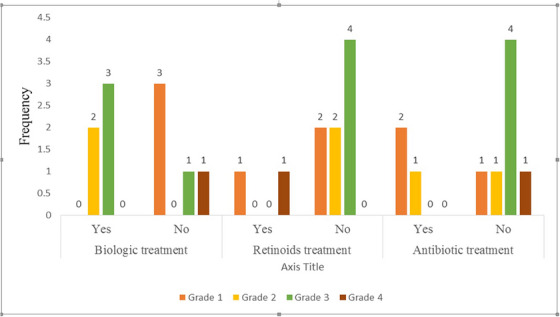
Distribution of different types of treatments according to disease grading score.

Among the understudied axillary hydradenitis suppurativa cases, six (60%) patients were males and the patients’ mean (Standard deviation) age was 30.10 (6.75) years old. Regarding the smoking status, three (30%) of cases had a history of cigarette smoking. Also, six (60%) and five (50%) of cases had a history of receiving antibiotics and Biologic treatments, respectively, and one patient had a history of surgery. The mean (Standard deviation) of disease duration was 4.80 (4.04) years. More descriptive information on the patient was shown in Table 1. At the time of intervention, most subjects received biological and antibiotics drugs, respectively (50% and 30%) (Table 2). The distribution of different types of treatments according to disease grading score is shown in Figure 1.

**TABLE 1:** Descriptive characteristics of patients with axillary hydradenitis suppurativa (*n*: 10).

Variables		
Gender	Female	4 (40)
	Male	6 (60)

		Mean (SD)
Age		30.1 (6.7)
BMI		31.3 (5.6)
Disease duration		4.8 (4.0)

		N (%)
Smoking	Yes	3 (30)
Grade	1, 6	0
	2	3 (30)
	3	3 (30)
	4	1 (10)
	5	3 (30)
Drug history	Antibiotics	6 (60)
	Steroids	1 (10)
	Retinoids	4 (40)
	Biologic drugs	5 (50)
History of Surgery	Yes	1 (10)
Current medication	Antibiotics	3 (30)
	Retinoids	2 (20)
	Biologic drugs	5 (50)

**TABLE 2:** Patients’ outcomes including satisfaction level and tolerability three months after the intervention and recurrence rate with a 6‐month follow‐up.

Current status		*N* (%)
Treatment Satisfaction level	Unsatisfied	2 (20)
	Medium	2 (20)
	Good	3 (30)
	Excellent	3 (30)
Tolerance status	Yes	8 (80)
Recurrence status	No	4 (40)
	During the first 3‐month follow up	4 (40)
	During the second 3‐month follow up	2 (20)

### 3.2 Patients’ outcomes

**FIGURE 2:**
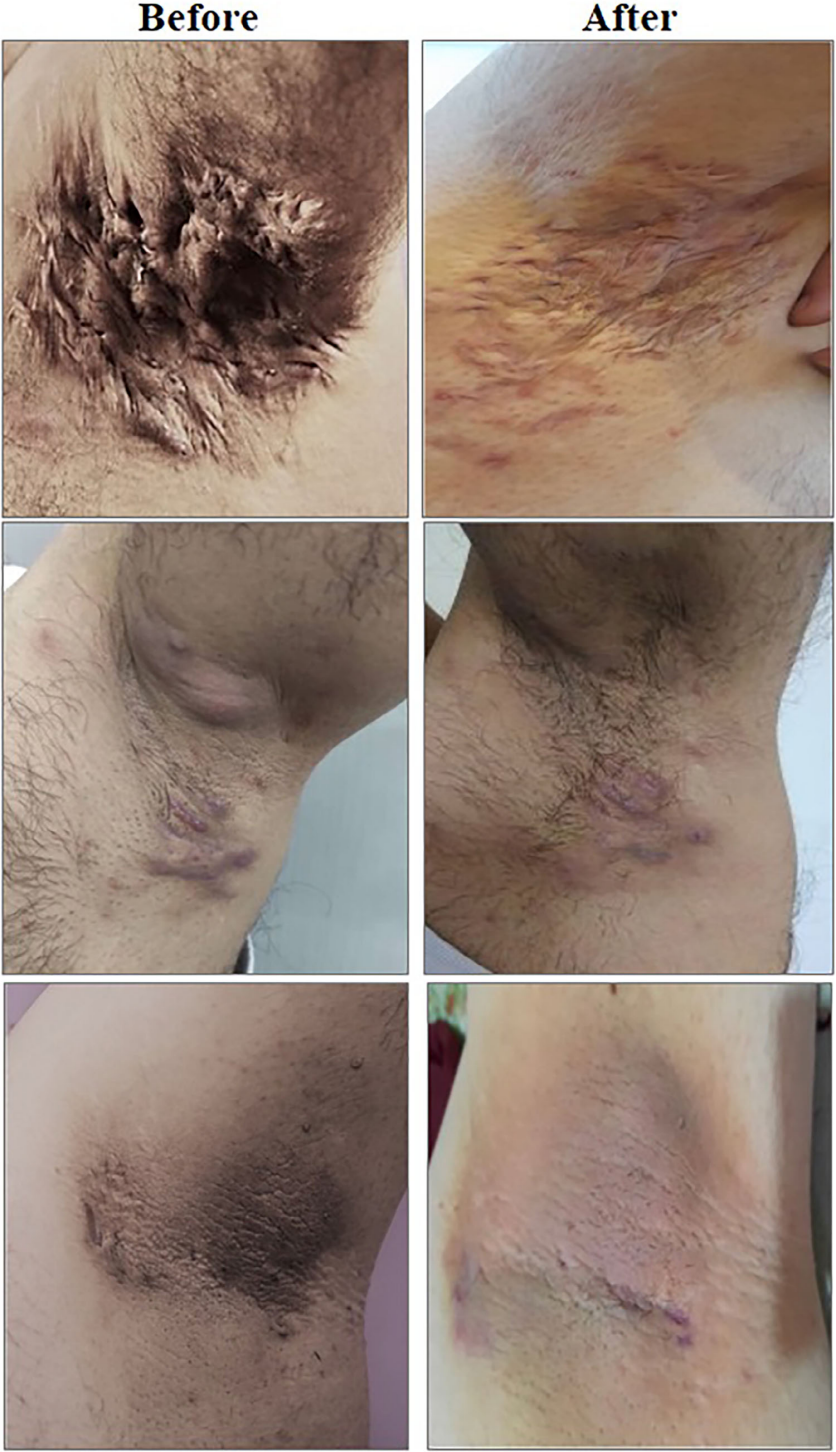
Clinical status of patients with hidradenitis suppurativa of the axilla treated with the Endo‐RF device. a: before the procedure, b: after a 6 months follow‐up.

The satisfaction level after the intervention among under‐studied cases was excellent and good in three (30%) and three (30%) of cases, respectively, however 20% of cases were unsatisfied. There was a significant difference in comparing the grading score of patients before and 3 months after receiving RF (*P*‐value: 0.01) and in nine (90%) of patients the grade of HS decreased (Table 3 and Figures 1, 2). Regarding tolerability (yes or no), eight (80%) of the patients who received the RF could tolerate it. We had no complication after the intervention and four cases had not recurrence during a 6‐month follow up (Table 2).

**TABLE 3:** Comparing the grading score of patients before and three months after the intervention.

Patient number	Before intervention	After intervention	*P*‐value
1	5	3	0.01
2	2	1	
3	3	2	
4	5	3	
5	5	3	
6	4	3	
7	3	2	
8	3	4	
9	2	1	
10	2	1	

## 4 DISCUSSION

Hidradenitis suppurativa is a chronic and debilitating inflammatory disorder that leads to poor quality of life and high burden.^4^, ^10^, ^11^ According to current guidelines, several medical and surgical options are available for the treatment of HS however; they may not always result in satisfactory outcomes.^1^, ^12^ Due to relapse following discontinuation of most medical options and severe side effects and high recurrence rate with surgery methods, successful management of HS remains controversial and new therapeutic options are needed.^6^, ^13^ In this non‐randomized, single‐blinded clinical trial, we evaluated the efficacy of radiofrequency in the treatment of 10 patients with refractory axillary hidradenitis suppurativa. After a three‐month follow‐up period, patients’ HS‐PGA scores improved significantly and the HS‐PGA scale showed a reduction in 90% of the cases. In addition, 80% of the patients reported moderate to excellent satisfaction during follow‐ups. Despite the efficacy of RF in three‐month follow up, the disease recurrence in 9‐month follow up reached 60% which highlights the importance of repeating periodic therapy session with RF. Due to our recurrence rate results in our patients’ follow up visits, repeating RF therapy sessions each 6 month seems to be appropriate for controlling the HS symptoms.

In a three‐arm parallel design randomized clinical trial, the efficacy of combination therapy with Intense Pulsed Light (IPL) and RF in the treatment of HS was compared to stand‐alone therapy with IPL or RF. The results demonstrated that combining RF with IPL can improve treatment effectiveness in terms of reducing active lesion counts. Furthermore, compared to the IPL group, patients in the IPL+RF and RF groups had a remarkably greater in Dermatology Quality of Life Index (DLQI) at 12 weeks.^2^ In another study, a 17‐year‐old boy with a history of HS was successfully treated with a non‐ablative RF device. He had undergone several therapy options, including antibiotics, topical formulations, retinoids, topical antibacterial agents, and intralesional triamcinolone, during a 6‐year period since the onset of his disease, with limited to no improvement. The patient then received three sessions of non‐ablative RF over a four‐month period, which resulted in significant improvement in symptoms, a reduction in new lesions, an improvement in the quality and duration of the lesions, a decrease in the pain associated with the onset of the lesions, and a shorter time to full resolution of new lesions.^3^ A few studies have reported efficacy of fractional micro needling radiofrequency (FMR) in successful management of HS.^2^, ^3^, ^5^ FMR is a method which utilizes microneedles to convey energy directly to the dermis.^5^ Through FMR; energy is delivered to dermis effectively while epidermis which plays a key role in quick healing remains intact.^5^, ^14^ This FMR feature leads minimizing the complications of patients following the procedure.^5^ However, the literature on this subject is still limited and the numbers of patients studied have been mostly small. A study by J.H. Yang et al. which investigated the therapeutic effect of FMR on clinical and pathological aspects of HS patients showed that number of HS inflammatory lesions will be reduced after approximately one month while reduction in HS‐PGA and modified Sartorius score (mSS) happens 6 months after the procedure.^5^ In addition to clinical improvements, histopathological studies revealed significant reduction in inflammatory markers including IL‐8, IL‐17, TNF‐α, TGF‐β1, neutrophil elastases, and MMPs^5^. In a study by V Madan et al. investigated the efficacy of carbon dioxide laser in recalcitrant severe hidradenitis suppurativa patients which were resistant to other medical or surgical therapies. The results found complete remission for one year or longer in roughly 80% of the cases. Furthermore, patients stated high levels of satisfaction in the study and except for two axillary scar contractures, no other main adverse events were reported.^15^ In addition to laser and light‐Based treatment methods, RF, and FMR, some other therapeutic modalities for treatment of HS such as electrosurgery, have also been studied.^7^, ^8^ In a study which surveyed the efficacy of electrosurgery in twelve grades 1 and 2 HS cases with electrosurgery, improvement was observed in more than 80% of the patients and lesions.^7^ There are several limitations in our study which should be addressed in future investigations. Firstly, the number of patients in our trial was small which can be resolved by multicenter studies. Secondly, the patients had been receiving different therapeutic options due to severity of the disease which may confound with the impact of studied therapeutic method. This limitation may be addressed by termination of other therapies before the start of the trial or comparing the patients with the same drug/treatment history.

## 5 CONCLUSION

Hidradenitis Suppurativa (HS) is a chronic and recurrent disease of the axilla and groin with inflammatory lesions. There is no definitive medication or intervention to cure the disease. RF is a safe and effective therapeutic approach for controlling axillary HS however to prevent the recurrence, periodic therapy sessions are needed.

### Study limitation

The number of patients can be considered as limitation of this study.

## AUTHOR CONTRIBUTIONS

All authors contributed to the preparation of data and the finalization of this article. All the figures have been produced by the authors of this article and are personal data. Elham Behrangi and Elaheh Lotfi made the idea, edited the proposal, registered the trial in IRCT and edited the final article. Azadeh Goodarzi and Najmolsadat Atefi wrote the proposal and edited the proposal until receiving ethical code. Elham Behrangi, Najmolsadat Atefi, Pardissadat Mireshghollah, Abbas Dehghani, Ali Zare Dehnavi, Masoumeh Roohaninasab, and Sara Dilmaghani managed the data and wrote the main draft of the article, and edited final article. AG revised and submitted the manuscript.

## CONFLICT OF INTEREST STATEMENT

The authors declare there is no conflict of interests.

## ETHICS STATEMENT

The authors obtained informed consent for publication of this study and all accompanying images. All of the collected information was kept confidential and analyzed without a specific name. The present participants in this project were adhered to all Helsinki ethical principles (IRCT20200901048586N1). This research was approved by the Research Council with the ethics code number IR.IUMS.FMD.REC.1401.005.

## TRIAL REGISTRATION

The trial was registered in Iranian Registry of Clinical Trials (IRCT) with the ethics code number IR.IUMS.FMD.REC.1401.005.

## Data Availability

The data that support the findings of this study are available from the corresponding author upon reasonable request.

## References

[1] Zouboulis CC , Desai N , Emtestam L , et al., European S1 guideline for the treatment of hidradenitis suppurativa/acne inversa. J Eur Acad Dermatol Venereol. 2015;29(4):619‐644. 25640693 10.1111/jdv.12966

[2] Wilden, S. , Friis M , Tuettenberg A , et al. Combined treatment of hidradenitis suppurativa with intense pulsed light (IPL) and radiofrequency (RF). J Dermatolog Treat. 2021;32(5): 530‐537. 31609667 10.1080/09546634.2019.1677842

[3] Iwasaki J , Marra DE , Fincher EF , Moy RL , Treatment of hidradenitis suppurativa with a nonablative radiofrequency device. Dermatol Surg. 2008;34(1):114‐117. 18053033 10.1111/j.1524-4725.2007.34025.x

[4] Jemec GB . Clinical practice. Hidradenitis suppurativa. N Engl J Med. 2012;366(2):158‐164. 22236226 10.1056/NEJMcp1014163

[5] Yang JH , Cho SI , Kim DH , et al. Pilot study of fractional microneedling radiofrequency for hidradenitis suppurativa assessed by clinical response and histology. Clin Exp Dermatol. 2022;47(2):335‐342. 34431555 10.1111/ced.14905

[6] Mehdizadeh A , Hazen PG , Bechara FG , et al. Recurrence of hidradenitis suppurativa after surgical management: a systematic review and meta‐analysis. J Am Acad Dermatol. 2015;73(5 Suppl 1):S70‐S77. 26470621 10.1016/j.jaad.2015.07.044

[7] Aksakal AB , Adişen E , Hidradenitis suppurativa: importance of early treatment; efficient treatment with electrosurgery. Dermatol Surg. 2008;34(2):228‐231. 18093196 10.1111/j.1524-4725.2007.34042.x

[8] Lyons AB , Townsend SM , Turk D , Narla S , Baah N , Hamzavi IH . Laser and light‐based treatment modalities for the management of hidradenitis suppurativa. Am J Clin Dermatol. 2020;21(2):237‐243. 31845121 10.1007/s40257-019-00491-1

[9] Carruthers J , Fabi S , Weiss R . Monopolar radiofrequency for skin tightening: our experience and a review of the literature. Dermatol Surg. 2014;40 Suppl 12:S168‐S173. 25417570 10.1097/DSS.0000000000000232

[10] Gill L , Williams M , Hamzavi I . Update on hidradenitis suppurativa: connecting the tracts. F1000Prime Rep. 2014;6:112. 25580266 10.12703/P6-112 PMC4278191

[11] Wolkenstein P , Loundou A , Barrau K , Auquier P , Revuz J . Quality of life impairment in hidradenitis suppurativa: a study of 61 cases. J Am Acad Dermatol. 2007;56(4):621‐623. 17097366 10.1016/j.jaad.2006.08.061

[12] Marasca C , Annunziata MC , Napolitano M , Fabbrocini G . Unconventional therapies for hidradenitis suppurativa. Expert Rev Clin Pharmacol. 2018;11(9):879‐887. 30136871 10.1080/17512433.2018.1509706

[13] Falola RA , DeFazio MV , Anghel EL , Mitnick CDB , Attinger CE , Evans KK . What heals hidradenitis suppurativa: surgery, immunosuppression, or both? Plast Reconstr Surg. 2016;138(3 Suppl):219S‐229S. 27556765 10.1097/PRS.0000000000002671

[14] Hantash BM , Renton B , Berkowitz RL , Stridde BC , Newman J . Pilot clinical study of a novel minimally invasive bipolar microneedle radiofrequency device. Lasers Surg Med. 2009;41(2):87‐95. 19226570 10.1002/lsm.20687

[15] Madan V , Hindle E , Hussain W , August PJ . Outcomes of treatment of nine cases of recalcitrant severe hidradenitis suppurativa with carbon dioxide laser. Br J Dermatol. 2008;159(6):1309‐1314. 19036028 10.1111/j.1365-2133.2008.08932.x

